# Embracing multiple stakeholders’ perspectives in defining competent simulation facilitators’ characteristics and educational behaviours: a qualitative study from Denmark, Korea, and Australia

**DOI:** 10.1186/s41077-022-00240-1

**Published:** 2023-01-09

**Authors:** Margrethe Duch Christensen, Doris Østergaard, Søren Stagelund, Leonie Watterson, Hyun Soo Chung, Peter Dieckmann

**Affiliations:** 1 Copenhagen Academy for Medical Education and Simulation (CAMES), Copenhagen, Denmark; 2 Department of Anaesthesiology, Nordsjællands Hospital, Hillerød, Denmark; 3 Department of Clinical Medicine, University of Copenhagen, Copenhagen, Denmark; 4 Department of Emergency Medicine, University Hospital of North Norway, Tromsø, Norway; 5 Sydney Clinical Skills and Simulation Centre, Sydney, Australia; 6 Department of Emergency Medicine, Yonsei University College of Medicine Korea, Seoul, South Korea; 7 Department of Quality and Health Technology, University of Stavanger, Stavanger, Norway; 8 Institute for Public Health, Copenhagen University, Copenhagen, Denmark

**Keywords:** Simulation-based learning, Facilitator, Instructor, Educator, Qualitative research, Cultural differences, Simulation training, Competence, Capabilities, Characteristic

## Abstract

**Background:**

Simulation-based learning (SBL), used for achieving various learning goals, is spreading around the world. However, it is still open, to what extend SBL needs to be adapted to local cultures. This study aimed to explore how various stakeholder groups perceive what constitutes a competent simulation facilitator across three different countries.

**Methods:**

We conducted an interview study with learners, facilitators, and facilitator trainers. Semi-structured interviews with 75 participants underwent content analysis. Participants were recruited from Denmark, Korea, and Australia. Interviews focused on characteristics of simulation faculty, as well as educational behaviours. Interviews were audio-recorded, translated to English, transcribed, and content analysed by inductively developing codes using the Nvivo software. In the first coding round, each interview was treated separately. In the analysis round, the individual codes between countries and stakeholder groups were compared to identify similarities and differences.

**Results:**

Our study shows high demands for the simulation facilitator role. A competent simulation facilitator should possess the following characteristics: (1) subject matter expertise, (2) personal approach and traits, (3) self-awareness and reflection, and (4) communication skills. Educational behaviours comprised (1) supporting a safe learning environment, 2) working goal-oriented with the course, (3) engaging before the course with preparation, (4) leading scenarios, and (5) facilitating debriefings. Comparative analysis showed similar wishes towards simulation facilitators from the different stakeholders in different countries, though the same terms might mean different details in the various settings.

**Conclusions:**

These findings offer guidance for learning needs analysis and the establishment of faculty development programmes. The study also shows that the personal characteristics are an important aspect of the facilitator role above and beyond displaying educational behaviours.

**Supplementary Information:**

The online version contains supplementary material available at 10.1186/s41077-022-00240-1.

## Introduction

The use of simulation-based learning (SBL) for achieving various learning goals is spreading around the world. In countries with a shorter tradition for SBL, many instructor courses for facilitators and simulation faculty are conducted by groups from countries with longstanding practice of SBL, for example, many European countries, North America and Australia [1, 2]. The essential role of facilitators in participants’ learning in SBL, especially during debriefing, is recognised across settings [1–7]. The Society of Simulation in Healthcare defines a facilitator as an “individual that helps to bring about an outcome (such as learning, productivity, or communication) by providing indirect or unobtrusive assistance, guidance, or supervision” [8]. Their actual tasks and role definition vary across countries, but typically involve, for example, setting and explaining the frames for a conversation (e.g. the goal and the basic methodological steps), guiding the conversation (e.g. with questions or summaries), or monitoring the group processes and intervening, where necessary. Facilitation is its own practice field with a long tradition outside of healthcare, though a discussion of the historic details is beyond the scope of this paper [9].

Using models for SBL in different cultures without adaptation (e.g. concepts and courses) may prove to be problematic [10, 11]. There is a risk that these concepts and courses do not correlate to the local norms, values, and beliefs. For example, Asian students are described as quieter and as expecting a teacher-centred style of instruction, whereas Australian students are found to be vigorously engaged in the discussions expecting a more learner-centred style of instruction [12–14]. A recently published study by Ulmer et al. found considerable differences in the description facilitators from different cultures offered, regarding how they lead a debriefing [11]. Facilitators in Korea have described how the debriefings are mostly teacher-led and tend to be more of a lecture format, as participants are quiet during the debriefings [15]. A study by Wong indicates that although Asian students are used to teacher-centred learning, this does not mean it is their preferred way of learning [16]..

Little is known about what constitutes a competent simulation facilitator in the eyes of different stakeholders in different countries. Note that we refer to the more overarching, holistic understanding of being competent, not to the fine-grained view and description of different competencies. We use “competent” similar to having the capability for the role, comprising more stable traits and personal preferences of being, as well as various knowledge and skills domains. We need research about the basic characteristics and behaviours simulation facilitators should have to maximise their learners’ learning process. Knowledge about what is expected of facilitators in different contexts, what is seen as “normal” or “good” is important for different reasons. This knowledge can guide the construction of aims and objectives of faculty development programs. It can guide about, how a competent faculty should “be” and how they should act. This knowledge can help in defining clear criteria for feedback and also help the individual facilitator to form and reflect upon their professional identity. When facilitators become more reflective about their role, we assume that they subsequently can better increase the learning experience for learners.

This study aimed to explore how various stakeholder groups perceive what constitutes a competent simulation facilitator across three different countries. This research, therefore, addresses two questions:

1. Which characteristics and educational behaviours do learners, facilitators and facilitator trainers expect from competent simulation facilitators?
2. How do these expectations on characteristics and educational behaviours of competent simulation facilitators vary across different countries?

## Methods

### Approach and paradigm

This study is interview-based and was undertaken at the Copenhagen Academy for Medical Education (CAMES) in Denmark, the Sydney Clinical Skills and Simulation Centre (SCSSC) in Australia and the Clinical Simulation Centre (CSC) Yonsei University College of Medicine in Seoul, South Korea. We applied a constructivist qualitative methodology to answer the research questions as we think this framework is especially suited for a study investigating debriefing practices in different countries [17].

### Sampling

We interviewed three groups of stakeholders regarding their expectations of simulation facilitators: learners, facilitators, and facilitator trainers. To obtain a broad perspective on the research questions, we invited learners from a variety of full-scale simulation-based courses in emergency medicine and anaesthesiology. The learners included physicians, nurses and paramedics, whose experience ranged from newly graduated to specialist level practice. All learners had prior experience as simulation participants from at least one simulation course. The interviewed facilitators were teaching in a broad range of courses using SBL, though these were predominantly team-oriented care for critically or acutely ill patients courses. Facilitator trainers were involved in faculty development courses in various constellations and all had several years of experience with simulation and facilitator training.

Invitation to participate in the study was based on convenience sampling (determined by previous collaborations) methods and extended to participants who attended courses at the three centres. There were logistical reasons for this approach, namely, working with facilitators that were “reachable” with the resources of this project. However, we also selected the partners with the aim of obtaining a geographical spread. Transversing the continents of Europe, Asia and Australia meant we could include insights from diverse cultures in different parts of the world. We used Hofstede’s definition of culture as the conceptual framework for this study, building on our previous work [10, 11]. Hofstede defines culture as “the collective programming of the mind that distinguishes the members of one group or category of people from others [18].

### Ethical considerations

The study was approved by Human Research Ethics Committee Sydney NSW (1305-162M). Danish Law exempts this type of study from ethical approval as it does not involve patients. Participants consented to participate after being informed about the nature of the study, the plans for publication and that they could withdraw from participation at any time. All analysis was conducted on an anonymous basis. Based on the nature of the study focus, we did not anticipate any negative effects for participants.

### Setting

Semi-structured group interviews were conducted with two to six participants. Each group consisted of either learners, facilitators, or facilitator trainers, respectively. The group format was chosen to stimulate the discussion, aiming for breadth of content, whilst accepting that this may comprise the depth of the interviews.

### Data collection

All study participants were asked the same set of questions, with additional questions for facilitators and facilitator trainers. The interviewer could explore participants’ statements for further clarification. The interview guide (Additional file 1: Appendix 1) was developed by SS and DOE and tested in two pilot interviews performed by SS.

The interviews in Korea were conducted in Korean, the interviews in Australia in English, and the interviews in Denmark in Danish.

### Researcher characteristics

Four experienced interviewers conducted the interviews. DOE, MDC, and SS conducted the Danish interviews and MDC conducted the Australian interviews. We consider the Danish interviewer as fluent in English, so that she could capture (most of) the nuances in the Australian English, as by the time of the interview conduct, she lived in Australia already several months. All authors consider themselves as proficient in English, to be able to analyse the English transcripts. A Korean staff member from the Department of Medical Education conducted the Korean interviews and wrote their summaries for further processing. This staff member holds a PhD within the area of education, had experiences with conducting qualitative research and interviews and felt confident to produce the summaries. This staff member was selected to reduce the power distance between the participants and the interviewer, as she was not in a work or educational relationship with the participants. Those the core writing team for this article, (MDC, DØ, PD, SS) are either native Danish speakers or lived in Denmark for more than 10 years and consider themselves able to handle the Danish transcripts and able to translate them into English for further processing. During the data processing, all researchers discussed the interpretation of the findings repeatedly, to reduce the challenges that lie in working in several languages [19, 20].

### Data processing

Table 1 provides an overview of the analysis steps. The interviews were audio-recorded and transcribed verbatim for the Australian and Danish interviews in English and Danish, respectively. The Korean interviews were audio-recorded and transcribed verbatim in Korean. A summary of the Korean transcripts was translated into English. The interviews lasted from 10 min (learners) to 75 min (facilitator trainers), depending on the number of participants and the study population. The transcribed interviews were imported to Nvivo version 11, which was utilised to assist in coding the transcripts.

**Table 1:** Overview of the analysis method

Analysis stages	Task
Stage 1	MDC read the Australian transcripts to get a general sense of the content with an open mind not yet marking any text elements. Emerging themes that described behaviours and characteristics of simulation instructors were identified and noted in a separate file for note taking.
Stage 2	MDC read the Australian transcripts a second time. Now meaningful units describing the characteristics (being) and behaviours (doing) for each group (trainees, instructor, and instructor trainers) were identified, coded, and discussed with PD. The codes closely reflected the spoken words of the participants.
Stage 3	The codebook was further refined as MDC performed a systematic content analysis of each code group, identifying subgroups and creating of a systematic condensation of the meaningful units within each code group into a new text condensate. The codebook was again discussed with PD.
Stage 4	Stage 1–3 was repeated for the Danish and Korean Interviews. It was not possible to create a text condensate from the Korean interviews due to the transcription method.
Stage 5	DOE, MDC, and PD went through all the codes from the all the interviews in one pool and organised them in main themes and sub themes (classification of codes).
Stage 6	DOE, MDC, and PD went through the codes from each study group in each country and compared for similarities and differences.
Stage 7	The results was reviewed by LW, SS, and Chung and final adjustments were made based on their input.

### Content analysis

We performed a descriptive analysis of the interviews staying as close to the data as possible, by using a language similar to the participants’ own language [17, 21–23]. For example, did several participants call the facilitators ‘instructors’—we kept the terms that they used in those quotes, but use the term “facilitator”, when we refer to these people, outside of quotes.

Initially, MDC read the Australian transcripts, without marking any text elements to establish a general sense of the content. Emerging themes that described behaviours and characteristics of simulation facilitators were identified and noted in a separate file for notetaking. Next, MDC read the Australian transcripts a second time. At this point, meaningful units describing the characteristics (being) and behaviours (doing) for each group (learners, facilitator and facilitator trainers) were identified, coded and discussed with PD. The emerging themes closely reflected the spoken words of the participants and were discussed amongst the research team. The content analysis was an iterative process, concluding when all members agreed on the sorting of codes and material. MDC, DOE and PD performed a comparative analysis across groups and countries, which was later discussed with the other authors. We analysed the perspective of the simulation facilitators across all countries as a starting point, as they are the focus of this research. We then compared the perspectives of learners and facilitator trainers to the perspectives of the facilitators, each across the different countries. We identified the common themes across the stakeholder groups. Those elements that could not be identified for all stakeholder groups were identified as discrepancies. Finally, the themes mentioned by the learners, the facilitators and the facilitator trainers across countries were countries were compared in order to describe the agreements and discrepancies.

## Results

Twenty-four group interviews were conducted across the three countries, with 23 participants from Australia, 36 from Denmark and 16 from Korea. Participants comprised 59 physicians, 15 nurses, and one paramedic (Table 2). We identified two broad areas for facilitator competence based on our content and comparative analysis: *facilitator* characteristics (Table 3) and *facilitators’ educational behaviours* (Table 4). Characteristics are in the realm of “being”, they represent attitudes and beliefs that the individual holds—those elements that an individual would describe, when asked about reasons behind behaviours. Behaviours are in the realm of “doings”. They are noticeable manifestations, typically verbal and non-verbal actions—saying things, doing things. Characteristics and actions do not have a one-on-one relation, but are connected and at times their distinction is more analytical than practical. Note that the behaviours described in table do contain not pure observable behaviours, but also interpretations of the effect of such behaviours. Consider, for example: “Creates a safe learning atmosphere”. There are many possible observable actions in this line and room for interpretation. What some participants might consider as helpful for the learning atmosphere, others might find disturbing—also because of possible differences in the national cultures. And yet, we find the constructs of being (characteristics) and doing (behaviours) helpful constructs for the given context. A lot of the characteristics can only become noticeable via actions, at times only over time. On the other hand, one might have characteristic, for example, being approachable in general, but not showing this on this specific day with this specific person. Characteristics can also be seen as the potential a person has and the behaviours, how this person elects to substantiate such characteristic. We see the characteristics not as fixed traits, but as tendencies of a person for certain preferences of behaviour.

**Table 2:** Overview of study participants per country

	Australia	Denmark	Korea	Number of participants
Learners (L)	11 physicians	22 physicians	5 physicians 3 nurses	41
Facilitators (F)	3 physicians 2 nurses	4 physicians 4 nurses	3 physicians 1 nurse	17
Facilitator (FT) Trainers	6 physicians 1 nurse	2 physicians 3 nurses 1 paramedic	3 physicians 1 nurse	17
Country	23	36	16	75

**Table 3:** Facilitators’ characteristics as described by all groups in all countries

Subject matter expertise	Personal approach and traits	Self-awareness and reflection	Communicative qualifications
• Has the necessary knowledge and/or clinical experience • Understands the process of Simulation • Is technologically competent • Is formally trained in simulation-based learning • Is credible	• Is approachable, kind and positive • Is open-minded and curious • Is charismatic • Is enthusiastic • Has a good sense of humour • Is creative and flexible • Is observant • Is structured and organised • Is able to empathise • Is acceptable towards others/accepts others • Has respect for the trainee and team members • Is non-judgemental • Is a team player • Shows leadership	• Is confident in one-self • Is humble • Accepts fallibility • Is open for dialog and recognises own limitations • Is willing to accept feedback/accepts feedback • Is willing to learn as well • Is open for supervision • Reflects on his/her own experience	• Has good communication skills • Reads and uses non-verbal communication • Communicates knowledge and ideas • Is a good listener

**Table 4:** Facilitators’ educational behaviours as described by all groups in all countries

Engages before the course	Supports a safe learning environment	Works goal-oriented with the course	Leads the Scenario	Facilitates the debriefing
• Prepares for the course • Develops good scenarios • Understands learner needs	• Understands the trainee • Pitches the right level • Creates a safe learning atmosphere • Maintains confidentiality • Builds a rapport • Builds a good group dynamic • Encourages the participants • Makes people feel comfortable • Handles different participants • Clearly communicates the frames • Gently pushes the participants out of their comfort zone • Provides psychological safety • Handles emotional reactions • Handles participant anxiety • Considers feelings • Addresses difficult issues • Does not criticise • Identifies good performances • Is able to understand different personalities • Is good at sensing moods	• Manages time well • Sticks to goals and objectives • Adapts to challenges • Is capable of making decisions during a course and a scenario • Maximises the learning opportunities • Participates actively during the course • Is engaged in the course • Uses clinical examples • Systematically evaluates students • Handles learners that underperform • Provides opportunity for repeating the training • Invites the learners to share reactions after the course • Learns new technology • Pushes boundaries on education	• Makes the simulation feel realistic • Handles difficulties with immersion • Runs a simulation with structure and flexibility • Pauses (interrupts) a scenario only when needed • Adjusts the scenario to the participants and the learning points • Is willing to role play • Gently pushes the participants out of their comfort zone	• Structures debriefing • Explores decision making processes • Facilitates reflection • Facilitates debriefing • Recognises and manages pitfalls • Is not being didactic • Facilitates group discussions • Gives constructive criticism • Facilitates more senior colleagues

In addition, we identified similarities and differences within each group of stakeholders in each country, from the comparative analysis (Table 5).

**Table 5:** Similarities and differences by groups and countries

**Differences within a group across all countries**		
Learners, all countries	Facilitators, all countries	Facilitator trainers, all countries
• Creates a safe learning atmosphere • Makes people feel comfortable • Runs the scenario without interruptions • Facilitates group discussion • Gives constructive feedback • Communicates knowledge and ideas well	• Facilitates group discussions • Gives non-judgemental feedback • Explores decision making process	• Leads different learners with different characteristics through the whole cycle of experiential and reflective learning.
Exemplary citations		
“Someone, who is able to write good scenarios. Should have basic medical knowledge. Should be able to make student emotionally and mentally safe. Provide comfortable environment (e.g. ice-breaking, giving enough time to get familiar with the environment). Should not make complicated scenarios. Must not lead students to experience bad patient outcome.” K13-FT “They can crack a joke and you need to make people comfortable.” AU2-L “So, I think to be able to kind of gently nudge somewhat reluctant participants to fully immerse themselves in it is also a good quality to have in an instructor. AU2-L	“So, you want the teacher to be somebody that recognises your mistakes, but doesn’t make you feel bad about them, so non-judgemental I’d call it.” AU2-F “Someone, who is trying to listen to the students carefully. Who is prepared well in advance and is able to run the scenario systematically and smoothly.” K9-F “Sometimes in the lectures during the courses, we have some topics […] like clinical decision making. […] And when we then have scenarios, where [participants] take many decisions, we sometimes do not relate at all what they just heard [in the lecture] and where participants might think ‘what is going on here?’ where there is not a real connection between the different parts. It is important that we describe such connections and that we build on each other’s work.” DK29-F	“Not sure, how to formulate that, but we are all trained professionals and have learned the same. But as persons are, we are very different. And maybe there is some super-smart people, who like to be “on”, whereas other prefer to basically stand in the corner as observers in all the scenarios.” DK-33-FT
**Differences for groups in each country**		
Australia, all groups	Denmark, all groups	Korea, all groups
• Is a good team player • Is humble • Has a good sense of humour • Is approachable • Is confident in oneself • Is Non-judgemental • Gently pushes the trainee out of their comfort zone • Facilitates group discussion	• Is enthusiastic • Is approachable • Has a good sense of humour • Runs a scenario with structure and flexibility • Creates a safe learning atmosphere	• Is formally trained in simulation- based learning • Is creative • Accepts others • Considers feelings • Is a good listener • Creates a comfortable atmosphere • Does not criticise
Exemplary citations		
“I feel much more likely to be focused in paying attention and taking away messages from people in the scenarios if I feel like, yeah, this person I think they give good anaesthetic. They know what they’re doing, they’ve read the recent evidence, they’re presenting information which makes sense which I can relate to in my clinical practice” AU8-L “That you [as facilitator] admit your fallibility I guess, and that might come up in a discussion about something or – so again they don’t feel like you’re trying to be seen as an infallible expert and everything, because that makes it easier for them to kind of learn and discuss things.” AU6-FT	“It is also important that facilitators signal that that they are open for questions, that it is a forum to ask questions and not just the lecture part and then ‘goodbye’.” DK1-L “So, you should really signal ‘I want this simulation here, I enjoy this as well – I am really happy to be in this situation together with you’.” DK27-F “I think also you need this basic curiosity, an interest in other human beings. I believe a lot of this you can learn, but I also think, you recognize quickly–also in experienced facilitators, who dig deeper during a debriefing, until they got, what they wanted to hear and then just proceed. Where you could have stayed a while longer with the topic, if you really would have been interested in what participants say.” DK33-FT	“As a student, I don’t like being pointed out about my mistakes during the debriefing session. Instructors could consider students’ feelings and try to talk without blaming. Without bias, instructors should be able to deliver objective facts or information.” K3-L “An instructor should have abundant experiences and specialized and deep knowledge. It would be good if an instructor has official certificate.” K6-L
**Similarities within a group across all countries and all groups**		
• Is knowledgeable • Has the relevant clinical expertise • Is adequately prepared • Manages time well • Is flexible		
Exemplary citations		
“The scenarios particularly will go off track and [you have to] work out how to bring scenarios back onto track and that’s a tough thing. […] There’s nearly always a way that you can save face or bring the scenario back where you want to go, but you need that flexibility in terms of what cue that’s not written into the scenario can we add at this point to get them back on track.” AU19-FT “Then I said ‘they’ve got to be able to be flexible and know how to utilise the resources and the people in the sim centre, not just come along and be passengers, but actually think about how does a course run well, not just how does my session run well that some people almost manage to do our course by not thinking about what needs to happen. It’s a logistical challenge running a course and people don’t–The good instructors are on to that.” AU19-FT		

The text below illustrates the different elements with exemplary citations. Tables 3, 4, and 5 provide an overview of the different aspects that were mentioned.

The codes for the citations describe the countries (AU: Australia, DK: Denmark; K: Korea), a running number for the participant in a country, and letters indicating their role (L: learners, F: facilitators, FT: facilitators trainer). We selected citations here for the more complex issues that were mentioned, less complex points (e.g. different techniques) are presented in the tables only.

### Facilitator characteristics

Facilitators’ characteristics were described by four overarching categories (see Table 3). The characteristics described emphasize how demanding the role of a facilitator can be—it is not only what facilitators do, but also the mindset and attitudes they bring to the session. The emphasis on being flexible and adaptable that is prominent throughout the interviews describes the need for and ability to balance the different, possibly contradictory characteristics and actions (e.g. being humble in some contexts and with some participants and possessing—and demonstrating- subject matter expertise in other contexts and with other participants). Note that we distinguished characteristics as the abilities, the potential that a person has, which may or may not be substantiated in a given situation.

Subject matter expertise described the familiarity of the facilitator with the content being addressed in the course (e.g. airway management, decision making, dilemmas in nursing). This includes theoretical knowledge, but also practical abilities—a general competence within the issues that are being taught.

“[Facilitators] need to have the subject matter sorted out – you simply need to know – what participants ask about.” DK27-F

The personal approach and traits comprise the style of interaction, the values that a facilitator stands in for and the way she or he is doing so. It addresses, how facilitators react when being approached by participants and what kind of interaction they themselves initiate. It also describes, how facilitators adapt to the different flow of each individual course in collaboration with others. These interaction patterns were perceived as rather stable over time and therefore, were described as traits.

“[…] you have to be able to be good at transferring information or techniques. And obviously, that requires both having the knowledge and skills and being up to-date yourself, but then also being able to relay them in a manner that different people can understand. And you have to be flexible enough to know that what works for one person might not work for another person. And you also have to be intuitive enough to know what an individual candidate might need.” AU12- F

“All those instructors here kind of seemed interested in finding out a bit about you as a person as well as a potential anaesthetist. […] And yeah, I think that they’re relatable people. They’re approachable. Relatable. You feel like you can ask them questions. They’re not gonna judge you or shoot you down for asking something that might be a bit silly. So, I think being approachable and relatable” AU8-L.

“I think respectful is a really good word actually, no matter where participants have come from, no matter what they do or what they’ve done in that simulation they’ve still got experiences that you don’t have and you can learn something from them just like they can learn something from you. And I think respect is a good thing to have, definitely” AU17-FT

“It takes time for the student to grow. Thus, instructors should be patient and give enough time to students with appropriate feedback. It is very important to be considerate, thoughtful, and also acknowledge student’s attitude, as well as believing in them.” K 14- FT

Self-awareness and reflection described the willingness and ability to (re-)consider ones approach, to assess advantages and disadvantages of one’s methods and approach. This includes a mental representation of one’s own strengths and weaknesses in regard to the content being taught, the methods, being used, and the personal approach one takes. The difference to the personal approach and traits lies in a more inward orientation and the involvement of cognitive, and emotional processes. In contrast, the personal approach and traits comprises more elements of interactions with others and more observeable actions.

“It is also important that a facilitator not only pretends to be confident, says something, where you think yourself: ‘no that can simply not be true. I know that—this is different’ and then the facilitator just insists on what he or she said – then they miss authority, competence, and trust.” DK-27-F

Communication skills were collected in a separate theme (and not as part of the personal approach), because they were described as central and because they focussed more on the technical side of communication—being able to explain issues in a way that makes it easy to grasp them, being able to listen.

“It is important that you during the simulation scenario are really alert and can actually hear what is being said. You need to avoid something like: ‘Hey – you did not ask for a blood gas analysis’ and then the participant says ‘I did ask for one!’, but the facilitator did not hear that and did not provide any values.” DK27-F

“And if about 70% of conversations is supposed to be non-verbal, you need to be really good at reading that non-verbal […] how hard to push people is completely based on non-verbal cues. So, if [participants] come out of a scenario you need to know how traumatized they were or whether they feel good or bad about that scenario and based on that you need to use your debrief accordingly.” AU14-L

### Facilitator educational behaviours

Study participants had high expectations towards facilitators on many levels, both in terms of the interaction with the content and participants, but also in relation to the management of the course. The detailed behaviours were sorted according to different phases of SBL in Table 4.

“…[facilitators] got to be able to be flexible and know how to utilise the resources and the people in the simulation centre, not just come along and be passengers, but actually think about how does a course run well, not just how does my session run well that some people almost manage to do our course by not thinking about what needs to happen.” AU19-FT

“You see, to be engaged in the course – to answer your mails around the course planning and yes, that you are interested in the role of facilitator. I think that is important to not only think about this one day, but also about the before and the after of the course – that you are interested in what the evaluations showed. […] That is engagement of facilitators for me.” DK34-FT

The work begins before the course with preparation, for example by collecting as much information as possible about participants.

“If the level of candidates is lower than expected, the effect of the training could be low. Thus, instructors should know the candidates’ background and could adjust or revise the training content or scenarios.” K7-L

Facilitators also need to be supporting a safe learning environment addressing the different challenges that individual participants might experience. This requires time and techniques to make sure that participants understand what simulation-based learning entails. It also requires to monitor how learners react to the simulations.

“Students come from diverse places and backgrounds making their responses different to the scenarios. […] Thus, plenty of time for pre-briefing, briefing, orientation and ice-breaking is needed to have familiarity with each other.” K4-L

“So, I think time structure is a good thing and I think debriefing structure is a good thing. […] Because there’s a lot of things they’re going to be feeling unsafe about during the course of the day so you gotta try and […] make them feel comfortable in some regards whether its physical, or whether its psychological, some level of comfort.” AU18-FT

“I just think, it is nice to always repeat, when we run simulations: […] what happens here, stays here. Even though we might feel it is a repetition, it is important to verbalize it” DK2-L

“I think that potentially you could hit a nerve or someone could be reminded of some critical, you know, horrific incident that they were involved in.” AU8-L

As simulation-based education should help participants to reach the aim and objectives of the event, it is important to not only make the event “safe” but also goal-oriented. Participants might need support in getting the key learning messages. This concerns technical and conceptual issues, as well as taking the lead for the events unfolding and the continuous development of the course.

“Being prepared well is important and diligence is necessary….Technical or system errors could occur thus s/he should always have alternative plans.” K10-F

“[…] time, oh I think, is important that you as instructor are able to coordinate a whole day. You need to stick to the agreed times for scenarios and debriefings. So that you can make the day work out, also in regards to the others in the course.” DK26-F

“You need to take charge of things, show up in time, actually you need to come earlier to a course to plan, to fine-tune, have time to arrange things. And you might also need to adjust some things, if it becomes clear, for example, that the scenario does not work well, or creates some problems, or somethings else. Then you need to go ahead and make changes.” DK27-F

One major task to help learners is leading the simulation scenarios which create situations that bring participants to a learning zone, where they are challenged to a matching degree. Facilitators need techniques to keep the scenario running and relevant for participants and for adjusting it dynamically to the action of participants.

“Well for me, a good simulation instructor is someone who’s not afraid to just gently push the participants just a little bit out of their comfort zone in terms of the scenarios that they have to handle and meaning – Out of the comfort zone, meaning that the scenario is just a little bit beyond what they would sort of be familiar with, but that’s important as it gives them the opportunity to learn and then afterwards, to give constructive criticism and assurance during the debriefing.” AU11-L

“I think that’s what makes a really good simulation instructor is someone who can drop those small cues and hints without loosing the immersion so that you still get to those learning points. It’s a skill”. AU14-F

A lot of the learning potential is harvested in the debriefing. Here the task is to facilitate a learning conversation that helps to integrate the experience from the scenario into overarching learning points and that takes the characteristics of the learners into account.

“I don’t know everything, and I need to have that in me as an instructor to know that I’m not always right and I still have things to learn and I’ll potentially be learning from my participants as well.” AU14-F

“The things I appreciated about today was [name] showing how there's more than one way to do something. ‘If you don’t do it just the supervisor’s way, then you're wrong’ is not what happened today….It reflects the real world that there's lots of greyness. Every patient’s different. You can make big mistakes, but there's a number of ways to do the right thing. And I think that’s a much more enjoyable learning experience.” AU3-L

“So they need to be able to facilitate discussions appropriately. Be able to open people up in a way. To be able to encourage people that might be more reserved.” AU7-L

### Similarities and differences across countries and groups

Table 5 describes the similarities and differences across countries and groups. The different groups of participants emphasised different aspects of facilitators characteristics they seek in facilitators; an example of which was the leadership style used in the context of debriefing in Korea. Korean facilitators wished other facilitators to be authoritative, while facilitator trainers and the learners, on the other hand, wanted facilitators to be less authoritative. Additionally, Australians thought it was good for a facilitator to expend time and energy on setting the scene and creating a learning atmosphere, for example, by talking about their own mistakes at the beginning of the course. In contrast, the Danish and Korean facilitators did not allocate much time to this.

Across all countries and groups, the competent facilitator should have the necessary subject matter expertise needed for the respective course and scenario. On a personal level, qualifications such as being flexible, humble, approachable, kind, enthusiastic, and a good communicator on many levels was essential. The facilitator should also be educationally able to create learning opportunities by creatively preparing and conducting simulation sessions, being able to adjust to the learners and the dynamics during the different phases of a simulation course (especially in adjusting scenarios and debriefings) and manage time well during a course.

In Korea, there was an emphasis on the educational credibility as an instructor:

“If the government has certificate program for simulation education instructor, then he/she should acquire it. At this point, however, Korea does not have any certification program. Thus, the society or certain institutions should develop the program, which should include basic course, tests, theory and practice, etc “K1-L.

In Australia, the focus was placed more on the processes, tasks, and abilities:

“[…] understanding of the process, what’s involved with the scenarios, etc.; also the mannequins, the streaming, and the debrief process. The clinical, the team factors, the individual factors that are all coming together.” AU14-F.

Another area, where differences between countries showed up was in the evaluation of learning processes and outcomes.

Korea: “Learning needs to measured systematically evaluation tools and checklists and when the students did not achieve the course objectives, the instructor should re-organise the training content and provide additional opportunities for the students to achieve the objectives.” K2-L

Denmark: “cannot let the trainees go home and think they did the right thing, if they had done something that was wrong - that is, something that could be dangerous for the patients in the hospitals” DK34-FT.

“If you have a participant who performs below – I mean really way below standards, then you need to inform the clinical supervisor. Or find out whom to inform.” DK28-F

Australia: “ [when there was a mistake in the performance] this is where .. flexibility comes in, and you need to remain calm and go, ‘Alright cool, this isn’t exactly what our learning objective was but I think this is something that these guys needs to learn instead’.“ AU17-IT

## Discussion

In this study, we have identified the characteristics and educational behaviours that simulation participants, facilitators, and facilitator trainers see as constituting a “competent facilitator” for SBL in Denmark, Korea, and Australia. We see similarities and differences in the expectation for this role across countries and stakeholder groups. This can be expected as different people have different views on virtually any question—what makes a “good” facilitator in the context in this study. The results of our study contribute to describe the range of the these differences—they are one piece in the puzzle of describing “normal” facilitator characteristics and behaviours. Our results describe the range of “good facilitators” in different countries and across different target groups and can help individuals and teams to find their own version of “good facilitators” by reflecting upon, which of the characteristics and behaviours are relevant for them and to what degree and in what way they implement them. The tables serve as potential guides for (self-)reflection, where facilitators can relate their own preferences to the overview: what is also my style, where am I different? They can also serve as potential targets for faculty development, where either in self-reflection or in a conversation, facilitators can identify, where they want to develop in what way. They are formulated as generic lists and each individual will relate more to some of the aspects than others. Based on the large variation between individuals, we did not formulate connections or priorities.

In all contexts, stakeholders wish for a competent and approachable human being, who strives to be prepared and help the learners achieve the goal of the session. Our results emphasize the role of facilitators’ characteristics across countries and supports that faculty development should include such aspects besides the training of using certain interaction techniques. Asking questions after the book can still be “wrong”, when the rapport between facilitator and learner is damaged, or if the facilitator mainly fulfils personal motives by asking it.

The current findings are very much in line, with other studies. A focus group interview study with medical students distinguished instructor skills (setting up the learning environment, teaching at the appropriate level, teaching technique, providing deeper context, and giving effective feedback) and attributes (enthusiasm, engaged, prepared, knowledgeable, patient, relational, transparent, and calm). While using different words (skills and attributes), the study also distinguishes between an angle aimed more at characteristics and one aimed more at behaviours—both showing overlap with the findings in the current study [24]. A book chapter conceptualized simulation as social event, where those involved interact with each other based on more or less defined rules [25]. The current paper qualifies some of these rules, in terms of the mutual expectations. We found systematic differences (and many similarities) across countries, as our group has postulated [10] and showed before [11]. Facilitators in different countries and with different levels of power distance, report different interactions with participants. In societies with high power distance, for example, facilitators would tend to speak more in a debriefing as opposed to countries with a low power distance. The influential professional standards by the Academy for Medical Educators in the United Kingdom [3] distinguish core values that educators should hold (e.g. respect for other people) and then describes five domains of educational activities (planning, conducting, and managing educational activities, performing assessments, and building on new sciences). The certification by the Society for Simulation in Healthcare, inspired by the professional standards from the United Kingdom, comprises similar criteria, comprising “professional values and capabilities”, “healthcare and simuation knowledge/principles”, “educational principles applied to simulation”, “simulation resources and environments” as relevant to achieve certification as simulation educators [26]. The International Nursing Association for Clinical Simulation and Learning (INACSL) describes standards for the professional integrity of simulation facilitators and uses four criteria for the description: “foster and role model attributes of profes-sional integrity at all times”, “follow standards of practice, guidelines, prin- ciples, and ethics of one’s profession”, „create and maintain a safe learning environment”, and “require confidentiality of the performances and scenario content based on institutional policy and procedures” [27]. Like these frameworks, our study emphasised the complex nature of the role as simulation facilitator [1, 26–28].

Very similar words were used by all participants in the interviews and yet, there seems to be some differences that are not always easy to grasp. The same term can be fulfilled in different ways in the respective cultures (consider, for example, different ways of paying respect between human beings) [29]. Simulation reflects aspects of the social practice of the countries in which it takes place [23, 30–32]. This implies that each written standard would need to be localised to the context in which it is applied. Such an adaptation is likely to be necessary not only across different countries, but also to other differences within a country, for example by profession or discipline. Our interviews can help to investigate how the different facilitators’ characteristics and educational practices play out in different contexts.

The more experienced the participants were, the more they focused on the value that facilitators create and less on their own personal position and actions within the sessions. The participants focused on how the facilitator adapts the procedures to help the different learners through the whole learning cycle of experience and reflection. Further, all participants referred not only to debriefing behaviours of facilitators but various activities that facilitators are involved in throughout a simulation-based course, from preparation to post-course evaluations.

For faculty new to SBL, aspiring to achieve the right balance of characteristics that facilitators should exhibit, such as subject matter expertise, empathy, truthfulness, and vulnerability, can be daunting. The results illustrate that the facilitator role is complex and needs to combine a wealth of different educational approaches and—maybe even more important so—a certain mindset, a way of interacting with participants, and a way of being authentic. In its essence, the difference can be described in terms of actions and mindset: is it enough that facilitators exhibit the right actions, or do we also wish for, or even require, such a mind-set? This question, in turn, relates to basic assumptions about learning and the roles of teachers and learners. In previous work, we discussed this difference with reference to behaviouristic and constructivist elements, of acting and being in an educational setting, and related them to the content and complexity of the learning objectives of simulation-based training [33, 34]. Our conclusion was that the more complex the learning objectives are, the more the constructivist approach seem to benefit the learning in the opinion of the participants interviewed. This current study supplements the findings of these studies by emphasising the cultural situatedness of SBL—its nature as a social practice [24, 30, 31]. The overall complexity can be captured only in fragments in short facilitator courses [35], but they can help attendees of such courses to start a life-long journey of experimentation and reflection [1].

## Study strengths and limitations

A key strength of this study was that we interviewed several groups of stakeholders from three different cultures.

A limitation was that we needed to interview different groups of people separately. Therefore, the differences might not show up in a prominent way, and we might underestimate differences. This might be even more so because participants used the same words in many contexts, but differences in the connotations of these words might not become apparent in the interviews. For logistical reasons, it was not possible to get a full transcript of the interview in Korea. The content analysis was performed after all the interviews were performed. Hence, we did not have the opportunity to explore emerging themes and differences in subsequent interviews. Thereby limiting our conclusions on specific cultural differences that were expressed in more subtle ways, such as participants using the same word but in a slightly different context.

The current study provides an overview of characteristics and behaviour in facilitators making them seen as “good and competent”, in general. The study does not provide guidance to the context-dependent mix the different elements. At times facilitators need to show more or less of the actions described. For example, they might adjust how much they facilitate and how much they need to instruct in a given situation, with a given learner, or in regard to a given discussion. Also, in the light of the characteristics they should foreground, the context plays a role. At times they might need to emphasize being humble, at times they might need to mark that they have subject matter expertise to improve the working relationship to their participants. Our work can enlarge the considerations that individuals take, when adjusting their role to a given situation. They would then still need fine tune the mixture in which they make use of the points discussed. We hope that this focus helps in deliberately fulfilling the facilitator role to optimise the learning opportunities for course participants.

There was a delay between conducting the study and publishing the results of about seven years, which is a limitation. We believe that we have a fair description of the viewpoints of our sample from the time in which we conducted the study. Values and practices are in constant flow. Our participants’ view may or may not have changed over time—with the current data material, we cannot say. Comparing the findings of our study with current reflections and discussions in our practice, we still find the underlying principles and overarching findings relevant. We believe that the results are still relevant, as they point to underlying principles and overarching findings.

The Hofstede model, used in the selection of the participating cultures, has been critiqued for several aspects. These include, for example, an oversimplification (e.g. the model summarizes across nations, not showing the variation within a nation) and the limited data foundation (the model was constructed based on data from employees in one company) [36]. There are also other models that describe culture with different dimensions, one of the most prominent being the Globe model [37]. Both models are critiqued for on the basis of several arguments, including their static nature [38]. Others have argued that such overarching models can still be useful, when making sure that, for example, the scoring of the cultural dimensions is not confused with “the culture” as such [36]. More recently, the very notion of culture was challenged and a focus on more fine grained practices is suggested as opposed to a general consideration of culture [30, 39–41]. Considering the different points, we decided to keep the Hofstede definition as our starting point, also because we worked with the model before, but we decided to focus on the investigation of practices and values in the different countries. In this way, we hope to combine the intuitive value of the Hofstede definition and model with context-sensitive data collection, analysis, and interpretation.

We find that the model in combination with our focus on practices and values helped in describing a differentiated picture of facilitator characteristics and behaviours. We cannot exclude that the similarities and differences that we described relate more to the different simulation traditions in the countries than to the different cultures. Having separate interviewers in the different countries could have introduced a bias in a way that one interview might (consciously or unconsciously) influence the interviewees along with his or her own preferences and wishes. We believe that our collaborative approach and frequent discussions in the research team counteracted such tendencies in the analysis phase. Given the complexity of our study with three different groups of participants (learners, facilitators, and facilitator trainers in three different countries), we refrained from an analysis that introduced profession as a variable. We think that the reflections by the groups of participants described here can form the basis for further investigations that would look more at the distributions of the issues we identified.

## Future research

A key issue for future research is a more detailed understanding of the different connotations that different people might have around certain terms (e.g. how do you express respect for the learner appropriately in different contexts?). Another interesting direction concerns an investigation into which differences between groups make a difference. In addition, it would be valuable to evaluate the influence of profession and experience level.

## Conclusion

This study demonstrates that a competent simulation facilitator within SBL constitutes a set of facilitator characteristics and educational behaviours and hence, emphasises the complexity of this role. The findings can contribute to learning needs analysis and setting up of faculty development programmes.

## Supplementary Information

**Additional file 1: Appendix 1.** Interview guide.

## Data Availability

The data is available upon reasonable request to the corresponding author.

## Abbreviation

SBL: Simulation-based learning
