# Supplementary material for: Embracing multiple stakeholders’ perspectives in defining competent simulation facilitators’ characteristics and educational behaviours: a qualitative study from Denmark, Korea, and Australia
Source: Adv Simul (Lond). 2023 Jan 9;8:1. doi: 10.1186/s41077-022-00240-1 (PMC9830838; doi:10.1186/s41077-022-00240-1)
Supplement: Supplementary file 1 — Additional file 1: Appendix 1. Interview guide. [file 41077_2022_240_MOESM1_ESM.docx]

**Appendix A – Interview guide**

| Initial questions   - What comes to your mind when I say "Good simulation instructor". - What comes to your mind when I say "Bad simulation instructor".   Additional Questions   - Are there other characteristics and behaviours you expect a good simulation instructor must have? - What characteristics and behavior must a good simulation instructor not have? - What pitfalls must a simulation instructor be aware of? - Are there other skills and qualities a simulation instructor must have?   Questions only for the interview of simulation instructors and simulation instructor trainers   - What else do you expect of a simulation instructor before, during and after completion of a simulation-based training? |
| --- |

The interviewers were allowed to probing questions, where they deemed that necessary. For example: You mentioned “team player”, could you please tell me more about that?
